# LIFE-COURSE SOCIOECONOMIC STATUS AND MORBIDITY AND MORTALITY IN THE MIDUS NATIONAL SAMPLE

**DOI:** 10.1093/geroni/igac059.1931

**Published:** 2022-12-20

**Authors:** Yanping Jiang, Li Chen, Samuele Zilioli

**Affiliations:** Rutgers, The State University of New Jersey, New Brunswick, New Jersey, United States; Augusta University, Augusta, Georgia, United States; Wayne State University, Detroit, Michigan, United States

## Abstract

Socioeconomic status (SES) is a well-established social determinant of health shaping the distribution of the burden of morbidity and mortality. In this area, a less understood topic is how life-course SES affects physical health. This study aimed to examine the effects of childhood and adulthood SES and social mobility on morbidity and all-cause mortality. Data were drawn from the Midlife in the United States study (MIDUS, N = 7,108, ages 25-74 years, 88.4% of White, 51.6% of female). Childhood and adulthood SES were assessed at baseline (i.e., 1995-1996). Mortality was tracked through June of 2018 (N = 1,425 deceased). The number of comorbidities was assessed at baseline and the third wave of MIDUS in 2013-2014 (N = 2,786). Results showed that lower childhood SES was associated with a larger increase in the number of comorbidities at the third wave of MIDUS and higher mortality risk (ps < .05). However, when including adulthood SES in the model, adulthood SES, but not childhood SES, was associated with morbidity and mortality (ps < .01). Childhood SES was indirectly associated with morbidity and mortality through adulthood SES (ps < .01). Participants achieving upward mobility (i.e., low childhood SES, high adulthood SES) had a smaller increase in the number of comorbidities and lower mortality risk (ps < .01) than those reporting persistent low SES in childhood and adulthood. These findings indicate the unique effect of adulthood SES on physical health and highlight the potential health benefits of upward mobility in middle-aged and older US adults.

